# Cord Blood Levels of Angiopoietin-Like 7 (ANGPTL7) in Preterm Infants

**DOI:** 10.1155/2020/1892458

**Published:** 2020-11-27

**Authors:** Xin Xia, Zhuxiao Ren, Longli Yan, Xuaner Zheng, Haoming Yang, Memnmeng Kang, Huiheng Yan, Zhicheng Zhong, Fang Xu, Jiayu Miao, Jianlan Wang, Yongsheng Li, Wei Wei, Jie Yang

**Affiliations:** ^1^Department of Neonatology, Guangdong Women and Children Hospital, Guangzhou Medical University, Guangzhou, China; ^2^Department of Clinical Genetic Center, Guangdong Women and Children Hospital, Guangzhou Medical University, Guangzhou, China; ^3^Guangdong Cord Blood and Stem Cell Bank, Guangzhou, China

## Abstract

**Objective:**

ANGPTL7 is a member of the angiogenin-like protein family. Compared to other members, ANGPTL7 is the least known. Recent studies have explored the relationship between ANGPTL7 and multiple pathological processes and diseases. However, there is no research about ANGPTL7 in neonates. This study was designed to investigate the concentration of ANGPTL7 in cord blood of preterm infants.

**Method:**

Singleton infants born in November 2017 to June 2019 in the study hospital were enrolled in the study. Maternal and neonatal clinical data were collected. ANGPTL7 levels in cord blood and serum on the third day after birth were measured by an enzyme-linked immunosorbent assay.

**Result:**

A total of 182 infants were enrolled in this study. Patients were categorized into two groups by gestational age (102 preterm, 80 full-term). ANGPTL7 levels in preterm infants were significantly higher than that in full-term babies (*t* = 15.4, *P* < 0.001). In multiple line regression analysis, ANGPTL7 levels independently correlated with gestational age (*β* = −0.556, *P* < 0.001). There is also no correlation between preterm outcomes and ANGPTL7 levels. Cord blood levels of ANGPTL7 were significantly higher than those in serum on the third day after birth (*t* = 13.88, *P* < 0.001).

**Conclusion:**

Cord blood ANGPTL7 levels are higher in preterm infants than full-term babies. The levels are independently influenced by gestational ages and attenuated significantly after birth. The underlying mechanism needs to be further studied.

## 1. Introduction

Preterm birth is a worldwide epidemic, and the global incidence of preterm birth is approximately 15 million every year [1]. Premature babies are at high risk for various complications, higher rates of hospital admissions, long-term neurodevelopmental disorders, and behavioral, social-emotional, and learning difficulties in childhood [2]. Among preterm infants, those with a gestational age of less than 33 weeks are at highest risk of mortality and morbidity and cause huge financial and emotional stress on families and society [3]. The occurrence of preterm birth is a complicated process; despite many risk factors have been reported, most premature infants have no clear risk factors. Thus, research on finding novel predictors for preterm delivery is of great significance in understanding specific mechanism of preterm birth and in devising effective treatments.

Angiopoietin-like proteins (ANGPTLs) are a family of secretory proteins that have a similar structure to angiopoietin proteins, consisting of eight members (ANGPTL1-8) [4]. All proteins are characterized with two domains: an N-terminal coiled-coil domain that mediates homo-oligomerization and a C-terminal fibrinogen-like domain that binds Tie2, except for ANGPTL8, which lacks the later domain [4]. However, the fact that ANGPTLs do not bind to the angiopoietin receptor Tie2 or Tie1 makes it possible to distinguish it from angiopoietins [5]. ANGPTLs have been proved to be involved in multiple pathophysiological processes, such as lipid metabolism, inflammation, angiogenesis, and cancer [6–12]. ANGPTL 7 is the least known in this family. Currently available researches have shown the role of ANGPTL7 in patients with diabetes, acute heart failure, and hypertension [13–15]. However, the role of ANGPTL7 in preterm infants has not been previously reported. In this study, we investigated the distribution of ANGPTL7 in neonates.

## 2. Method

### 2.1. Study Population

From November 2017 to June 2019, 182 infants born in Guangdong Women and Children's Hospital were included in this study. Inclusion criteria: (a) born in the study hospital; (b) singleton birth; (c) without congenital abnormalities; (d) the mother was negative for hepatitis B (HBsAg and/or HBeAg) and C virus (anti-HCV), syphilis, HIV (anti-HIV-1 and anti-HIV-2) and IgM against cytomegalovirus, rubella, toxoplasma, and herpes simplex virus; and (e) with written consent form.

### 2.2. Cord Blood and Serum ANGPTL7

A cord blood sample and a serum sample on the third day after birth were collected from every included infant if a signed informed consent form was obtained from the parent or guardian, centrifuged, and then, stored at –80°C. ANGPTL7 levels were measured using a human ANGPTL7 enzyme-linked immunosorbent assay (ELISA) Kit (ELAAB Science Inc., Wuhan, China) following the manufacturer's instructions.

### 2.3. Statistical Analysis

Statistical analysis of the data was performed using SPSS software version 20.0 (SPSS, Inc., Chicago, IL, USA). Baseline continuous variables were expressed as mean ± standard deviation or median (interquartile range). Categorical variables were represented by numbers (percentages). Independent samples *T* test was used to compare the quantitative variables between two groups. Chi-square was used to compare the qualitative variables between the two groups. Pearson/Spearman's correlation coefficients were estimated to determine associations between ANGPTL7 levels and variables. Multiple linear regression analysis was used to estimate the unique independent predictive contribution of neonatal/maternal on ANGPTL7. The variables' distribution characteristics were estimated with single sample Kolmogrov-Smirnov test. Statistics were considered to be significant when *P* value < 0.05.

## 3. Result

### 3.1. Baseline Characteristics

A total of 182 infants (102 preterm, 80 full-term) met the inclusion criteria and were included in the study population. 103 (56.6%) were boys, 33.5% preterm, and 23.1% full-term. Gestational age is 25-41 weeks, and birth weight is 750-4180 g; there were significant difference in gestational age, birth weight, delivery mode, asphyxia, maternal age, antenatal steroids use, and ANGPTL7 levels between preterm and full-term groups (Table 1). ANGPTL7 levels in preterm group were significantly higher than that in full-term group (7.99 ± 4.01 vs. 1.05 ± 0.39; *t* = 15.4, *P* < 0.001).

### 3.2. ANGPTL7 Levels in Cord Blood

**Figure 1:**
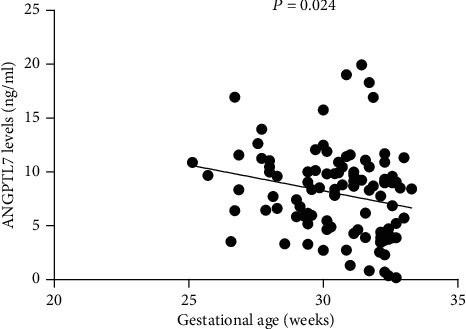
The correlation between ANGPTL7 and gestational age (^∗^*P* < 0.05).

We studied several maternal and neonatal factors to identify possible determinants of ANGPTL7 in cord blood. Cord blood levels of ANGPTL7 in preterm group were negatively correlated with gestation age (*r* = −0.223, *P* = 0.024, Figure 1). However, no correlations were observed between ANGPTL7 levels and neonatal and maternal variables in term group. In the overall univariate regression analysis, cord blood levels of ANGPTL7 were negatively correlated with gestation age (*r* = −0.743, *P* < 0.001) and birth weight (*r* = −0.707, *P* < 0.001). Maternal age (*r* = 0.241, *P* = 0.001), asphyxia (*r* = 0.164, *P* = 0.027), maternal hypertension (*r* = 0.264, *P* < 0.001), and antenatal steroid use (*r* = 0.449, *P* < 0.001) were positively associated with cord blood ANGPTL7 concentrations. We found no significant correlation between the cord blood levels of ANGPTL7 in infants and sex, delivery mode, and maternal diabetes. We used multiple regression analysis to control for potential confounding factors. The variables “birth weight” and “maternal age” were not introduced in the same model, as well as the variables “asphyxia,” “maternal hypertension,” and “antenatal steroids use,” due to collinearity. In multiple regression analysis, gestational age was the independent predictors of cord blood levels of ANGPTL7 (Table 2).

### 3.3. Cord Blood Levels of ANGPTL7 and Preterm Outcomes

To investigate the associations between ANGPTL7 levels and preterm outcomes, we compared the levels of ANGPTL7 between the affected and nonaffected groups in each complication (including BPD, NEC, IVH, ROP, and sepsis). The levels of ANGPTL7 were higher in infants who later developed BPD, NEC, IVH, ROP, and sepsis than those who did not, but the results all did not reach statistically significant differences (Table 3).

### 3.4. Serum Levels of ANGPTL7

**Figure 2:**
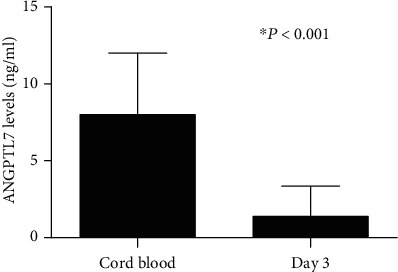
The comparation of ANGPTL7 levels in cord blood and serum on the third day after birth. Cord blood levels of ANGPTL7 (*n* = 102) vs. serum levels of ANGPTL 7 on day 3 (*n* = 57) (^∗^*P* < 0.05).

We also measured the ANGPTL7 levels in serum on the third day after birth. Since the consents for the collection of serum samples on day 3 were not obtained from all the parents or guardians, only 57 cord blood samples in preterm infants were finally collected. We found no maternal or neonatal characteristics have correlations with ANGPTL7 levels on day 3. However, serum levels of ANGPTL7 were significantly lower when compared with their levels in cord blood (*t* = 13.88, *P* < 0.001, Figure 2). This suggested that the levels of ANGPTL7 in preterm infants were significantly reduced after birth.

## 4. Discussion

In this study, we show for the first time the levels of ANGPT7 in preterm infants. Cord blood levels of ANGPTL7 are higher in preterm infants than that in full-term infants and have a negative correlation with gestational age. Serum levels of ANGPTL7 on day 3 of life were lower than those in cord blood. Collectively, our data shed the light on the expression pattern of ANGPTL7 in preterm infants.

ANGPTL7 is a newly discovered member of the angiogenin-like protein family. Previous studies have shown the biological function of ANGPTL7 in hematopoietic stem and progenitor cells [16, 17], trabecular meshwork cells [18, 19], cancer cells [20], and osteoblasts [21]. The important pathological processes involved in ANGPTL7 including inflammation [22], apoptosis [15], and angiogenesis [20, 23]. In our study, we found that ANGPTL7 levels were negatively correlated with gestational age in preterm infants, but not in term group. And the levels in preterm infants were significantly higher than term babies. This suggested us that ANGPTL7 possibly involved in the abnormal pregnancy process and a new biochemical marker for preterm delivery. Although the underlying causes of preterm birth are numerous, it is well known that inflammation represents a significant risk factor [24]. Qian and his colleague demonstrated that ANGPTL7 was a proinflammatory factor that promoted the inflammatory response in macrophages through the P38 signaling pathway [22]. The finding of Qian may support the negative association between ANGPTL7 and gestational age. In addition, future studies that compare the ANGPTL7 levels of preterm and term mothers may help us understand the underlying mechanism and identify women at high risk of preterm birth. Therefore, more research and evidence are needed in the future.

Hypertensive disorders of pregnancy were reported to be significant risk factors for preterm birth [25]. Previous study showed that ANGPTL7 was highly expressed in patients with hypertension, and downregulation of ANGPTL7 alleviated angiotensin II-induced hypertension and vascular inflammation [15]. In present study, we found no correlation between cord blood ANGPTL7 levels and pregnancy-induced hypertension or not. ANGPTL protein family has emerged as an important regulator of lipid metabolism [6, 11]. ANGPTL7 was also reported to be highly expressed in circulation and adipose tissue in obese patients and was positively associated with TG level. Similarly, ANGPTL7 was highly expressed in infants with maternal diabetes in our study, but no significant differences were observed when these two groups compared. And because of the unavailability of maternal data, correlation analysis was not done between ANGPTL7 and factors involved in lipid metabolism.

In this study, no significant differences were observed when ANGPTL7 levels were compared in each preterm outcome, even though the ANGPTL7 levels were higher in infants diagnosed with BPD, ROP, NEC, IVH, and sepsis than who was not. ANGPTL7 was originally discovered from corneal cells and named cornea-derived transcript 6 [26]. It was abundantly expressed in keratocytes, acted as an antiangiogenic protein to maintain corneal avascularity and transparency [23]. However, the role ANGPTL7 played in regulating angiogenesis in cancer was opposite to that in corneal vascularization. Parri and colleagues found that ANGPTL7 was a proangiogenic factor in human differentiated endothelial cells and can be specifically upregulated by hypoxia [20]. Angiogenesis is a complex biological process involving multiple aspects of preterm diseases as well. In preterm, retinopathy of prematurity (ROP) is a common complication that is characterized by retinal blood vessel growth arrest and retinal vascular growth disorganize caused by a large number of growth factors such as VEGF and IGF-1 [27]. Bronchopulmonary dysplasia (BPD) is a chronic pulmonary disease characterized by impaired alveolar and vascular development [28]. In the current study, the cord blood ANGPTL7 levels were both higher in ROP and BPD group than not but whether ANGPTL7 was involved as an antiangiogenic factor or a proangiogenic factor in the occurrence of ROP and BPD was unclear. In addition, since the observed negative relationship between ANGPTL7 and gestational age, exploring the role of ANGPTL7 in the occurrence of complications in preterm infants will be a new topic for future research.

## 5. Limitation

Our study has several limitations. First, sample size was limited. Only 182 infants were included in this study. The results could be caused by random error. Second, only 9 variables were included in the multiple regression analysis; we may have ignored other factors that related to ANGPTL7. Third, as there are few studies on ANGPTL7 by now, we cannot give a reasonable reason for its negative correlation with gestational age at present. Therefore, further studies with larger sample sizes are required to validate these results.

## 6. Conclusion

In conclusion, preterm infants have higher levels of ANGPTL7 than term babies. ANGPTL7 levels decreased during gestation, and attenuated significantly after birth. To the best of our knowledge, this is the first study that investigates the distribution of ANGPTL7 in preterm infant. However, there also remain some limitations. Thus, researches with larger sample size and explorations about the specific mechanism of the negative association between ANGPTL 7 and gestational age will be important areas of future research.

## Figures and Tables

**Table 1:** Neonatal and maternal characteristics compared between preterm and full-term neonates (^∗^*P* < 0.05).

Characteristics	Total (*n* = 182)	Preterm infants (*n* = 102)	Full-term infants (*n* = 80)	*P* value
Gestational age, weeks, mean ± SD	34.55 ± 4.67	30.55 ± 1.82	39.51 ± 1.04	<0.001^∗^
Sex, male, *n* (%)	103 (56.6%)	61 (59.8%)	42 (52.5%)	0.324
Birth weight, grams, mean ± SD	2262.91 ± 982.72	1454.22 ± 348.51	3294.00 ± 369.29	<0.001^∗^
Mode of delivery, vaginal, *n* (%)	103 (56.6%)	50 (49.0%)	53 (66.3%)	0.02^∗^
Asphyxia, *n* (%)	16 (8.8%)	16 (15.7%)	0 (0%)	<0.001^∗^
Maternal age, years, mean ± SD	30.92 ± 5.08	32.21 ± 5.57	29.29 ± 3.84	<0.001^∗^
Maternal hypertension, *n* (%)	41 (22.5%)	26 (25.5%)	15 (18.8%)	0.28
Maternal diabetes, *n* (%)	32 (17.6%)	22 (21.6%)	10 (12.5%)	0.111
Antenatal steroids, *n* (%)	47 (25.8%)	45 (44.1%)	2 (2.5%)	<0.001^∗^
ANGPTL7 levels, ng/ml, mean ± SD	4.94 ± 4.58	7.99 ± 4.01	1.05 ± 0.39	<0.001^∗^

**Table 2:** Multiple regression analysis model for cord blood levels of ANGPTL7 in preterm and full-term infants (^∗^*P* < 0.05).

Variables	*B*	95% CI for *B*	*P* value
Gestational age	-0.556	(-0.800, -0.311)	<0.001^∗^
Sex	0.341	(-0.595, 1.277)	0.473
Birth weight	-0.001	(-0.002, 0.001)	0.305
Mode of delivery	-0.019	(-1.041, 1.003)	0.971
Asphyxia	-1.228	(-2.944, 0.489)	0.160
Maternal age	0.002	(-0.099, 0.103)	0.964
Maternal hypertension	0.512	(-1.078, 2.102)	0.526
Maternal diabetes	0.169	(-1.044, 1.381)	0.784
Antenatal steroids	1.381	(0.191, 2.570)	0.053

*B*: unstandardized regression coefficient. Dependent variable: cord blood ANGPTL7 levels.

**Table 3:** Correlations between ANGPTL7 and preterm outcomes (^∗^*P* < 0.05).

Outcomes	ANGPTL7 levels		*P* value
	Yes	No	
BPD	9.51 ± 3.70 (*n* = 19)	7.64 ± 4.02 (*n* = 83)	0.067
IVH	8.83 ± 3.77 (*n* = 34)	7.57 ± 4.09 (*n* = 78)	0.133
ROP	9.35 ± 4.55 (*n* = 24)	7.57 ± 3.77 (*n* = 88)	0.058
NEC	8.20 ± 3.36 (*n* = 19)	7.94 ± 4.17 (*n* = 83)	0.802
Sepsis	9.50 ± 3.80 (*n* = 18)	7.66 ± 4.01 (*n* = 84)	0.079

BPD: bronchopulmonary dysplasia; IVH: intracranial hemorrhage; ROP: retinopathy of prematurity; NEC: necrotizing enterocolitis.

## Data Availability

Data will be provided by the corresponding author if required.
